# HBD3 Induces PD-L1 Expression on Head and Neck Squamous Cell Carcinoma Cell Lines

**DOI:** 10.3390/antibiotics8040161

**Published:** 2019-09-24

**Authors:** Maria Paula Gomez Hernandez, Amber M. Bates, Emily E. Starman, Emily A. Lanzel, Carissa Comnick, Xian Jin Xie, Kim A. Brogden

**Affiliations:** 1 Iowa Institute for Oral Health Research, College of Dentistry, University of Iowa, Iowa City, IA 52242, USA; paula-gomez@uiowa.edu (M.P.G.H.); emily-starman@uiowa.edu (E.E.S.); 2 Department of Human Oncology, University of Wisconsin School of Medicine and Public Health, University of Wisconsin-Madison, Madison, WI 53705, USA; ambates@wisc.edu; 3 Department of Oral Pathology, Radiology and Medicine, College of Dentistry, University of Iowa, Iowa City, IA 52242, USA; emily-lanzel@uiowa.edu; 4 Division of Biostatistics and Computational Biology, College of Dentistry, University of Iowa, Iowa City, IA 52242, USA; carissa-comnick@uiowa.edu (C.C.); xianjin-xie@uiowa.edu (X.J.X.); 5 Department of Periodontics, College of Dentistry, University of Iowa, Iowa City, IA 52242, USA

**Keywords:** PD-L1, HNSCC, SCC, carcinoma, oral cancer, defensin, HBD3

## Abstract

Human β-defensin 3 (HBD3) is an antimicrobial peptide up-regulated in the oral tissues of individuals with head and neck squamous cell carcinomas (HNSCC) and oral squamous cell carcinomas (SCC) and present in high concentrations in their saliva. In this study, we determined if HBD3 contributes to HNSCC pathogenesis by inducing programmed death-ligand 1 (PD-L1) expression on HNSCC cell lines. For this, SCC cell lines SCC4, SCC15, SCC19, SCC25, and SCC99 (5.0 × 10^4^ viable cells) were used. Cells were incubated with IFNγ (0.6 µM) and HBD3 (0.2, 2.0, or 20.0 µM) for 24 h. Cells alone served as controls. Cells were then treated with anti-human APC-CD274 (PD-L1) and Live/Dead Fixable Green Dead Cell Stain. Cells treated with an isotype antibody and cells alone served as controls. All cell suspensions were analyzed in a LSR II Violet Flow Cytometer. Cytometric data was analyzed using FlowJo software. Treatment with IFNγ (0.6 µM) increased the number of cells expressing PD-L1 (*p* < 0.05) with respect to controls. Treatment with HBD3 (20.0 µM) also increased the number of cells expressing PD-L1 (*p* < 0.05) with respect to controls. However, treatment with IFNγ (0.6 µM) was not significantly different from treatment with HBD3 (20.0 µM) and the numbers of cells expressing PD-L1 were similar (*p* = 1). Thus, HBD3 increases the number of cells expressing PD-L1. This is a novel concept, but the role HBD3 contributes to HNSCC pathogenesis by inducing PD-L1 expression in tumors will have to be determined.

## 1. Introduction

Head and neck squamous cell carcinomas (HNSCC) and oral squamous cell carcinomas (SCC) are neoplasms of oral tissues. Their onset and associated mutational profiles are often associated with prior alcohol consumption, tobacco use, and human papillomavirus infections [1,2,3]. In the most recent worldwide study in 2012, there were ~300,000 cases of HNSCC and SCC (2.1% of total cancer cases in the world, the sixth most common cancer by incidence worldwide) and ~145,000 associated deaths (1.8% of the world total) [4]. In the USA, there were 53,000 estimated new cases of HNSCC and SCC in 2019 (3.0% of new cancer cases) and 10,860 estimated deaths in 2019 (1.8% of all cancer deaths) [5]. The 5 years survival rate is 65.3% [5]. Only modest improvements in the survival rate are seen with chemoradiation, surgical resection, reconstructive methods, and biological treatments [6]. HNSCCs arise through the accumulation of genetic and epigenetic changes in genes acting in cancer-associated signaling pathways [1,7,8]. HNSCC cells produce a variety of immunosuppressive cytokines, chemokines, and biomarkers [9,10]. Among these is programmed death-ligand 1 (PD-L1) [10].

PD-L1 is a 33.28 kDa type I transmembrane protein expressed on the surface of immune and non-immune cells [11,12,13]. It is a co-inhibitory immune checkpoint protein that binds to the programmed death-1 (PD-1) receptor on T-cells [14]. The interaction of PD-L1 with PD-1 regulates the balance between co-stimulatory and co-inhibitory immune signals, maintains the breadth and magnitude of the immune response, maintains self-tolerance, prevents adverse autoimmune inflammatory events, protects the host from uncontrolled immune responses to pathogens, and prevents inflammatory tissue damage. Increases in PD-L1 expression can occur on SCC cells [8] as a result of mutations in tumor cell signaling pathways or exposure of tumor cells to inflammatory cytokines IL-1, IL-6, GM-CSF, IFNγ, TNFα, and VEGF [15,16,17,18,19] and the gamma-chain cytokines IL-2, IL-7, IL-10, IL-15, and IL-21 [20]. The latter group plays a role in peripheral T-cell expansion and survival. The presence of PD-L1 affects T-cell responsiveness in a quantitative manner [21]. A high level of PD-L1 expression increases impairment of T-cell survival and activity. Thus, a high level of PD-L1 expression on the surface of tumor cells inhibits the activation, expansion, and effector functions of T-cells [22,23,24] and helps SCC cells evade normal anti-tumor immune mechanisms.

HBD3 is a potent host defense peptide [25]. It has 45 amino acid residues and a monoisotopic mass of 5157.7 Da [26]. Its lysine and arginine residues gives it a strong positive charge [27]. HBD3 is expressed in mucosal epithelial cells and keratinocytes, including cells and tissues in the oral cavity [28,29]. It is present in gingival crevicular fluid and saliva.

HBD3 is a strong effector and regulator of innate immunity [30]. It has direct antimicrobial activity and can kill or inactivate Gram-negative bacteria, Gram-positive bacteria, yeasts, fungi, and viruses [26,29,31,32,33]. HBD3 can chemoattract mononuclear cells, phagocytic cells, immature dendritic cells, CD345RA expressing lymphocytes, and keratinocytes [34]. HBD3 can also induce the production of inflammatory mediators [35]. At low concentrations, HBD3 attenuates pro-inflammatory agonist-induced chemokine and pro-inflammatory cytokine responses; at high concentrations, HBD3 enhances agonist-induced chemokine and pro-inflammatory cytokine responses [36,37]. It can also regulate complement activation [38,39].

HBD3 enhances adaptive immunity [40]. It interacts with G protein-coupled receptors on immature dendritic cells, particularly CCR6 and induces NK cell activation, IFNγ secretion, and mature dendritic cell dependent cytolytic function [41].

In this study, we used SCC cell lines (Table 1). We report that HBD3 induces the expression of PD-L1 and this is a novel concept. However, to what extent HBD3 contributes to HNSCC and SCC pathogenesis by inducing PD-L1 expression in tumors will have to be determined.

## 2. Results

**Figure 1:**
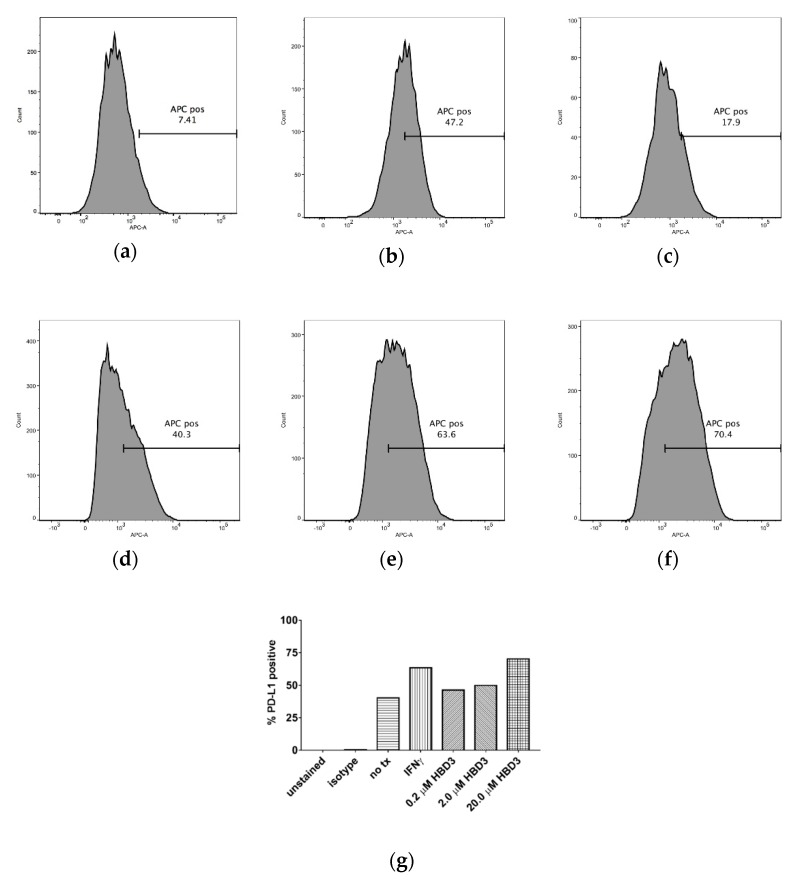
Flow cytometry analysis was used to show the effects of IFNγ and HBD3 treatment (tx) on the percent of cells with PD-L1 expression. In (**a**), 7.41% of SCC15 cells expressed PD-L1 (no tx). In (**b**), 47.2% SCC15 cells expressed PD-L1 after 0.6 µM IFNγ (tx). In (**c**), 17.9% SCC15 cells expressed PD-L1 after 20.0 µM HBD3 (tx). In (**d**), SCC99 cells expressed 40.3% PD-L1 (no tx). In (**e**), SCC99 cells expressed 63.6% PD-L1 after 0.6 µM IFNγ (tx). In (**f**), SCC99 cells expressed 70.4% PD-L1 after 20.0 µM HBD3 (tx). In (**g**), the effects of 0.6 µM IFNγ and 0.2, 2.0, or 20.0 µM HBD3 tx on the percent of SCC99 cells with PD-L1 expression can be seen. The percent of SCC99 cells with PD-L1 expression increased with increasing concentrations of HBD3. Unstained cells (e.g., unstained), cells stained with an isotype antibody (APC mouse IgG1 κ, BD Pharmingen, San Jose, CA) (e.g., isotype), and cells not treated with IFNγ and HBD3 (e.g., no tx) served as controls.

Bates et al. found that PD-L1 was present in differing concentrations on the surface of HNSCC cell lines in Table 1 by ELISA, and we summarized this information in Table 2 [10]. PD-L1 concentrations in cell lysates ranged from 79.67 to 539.79 pg/mL. In this study, we also found that PD-L1 is present on the surface of HNSCC cell lines by flow cytometry (Figure 1). The percent of SCC4, SCC15, SCC19, SCC25, and SCC99 cells with PD-L1 expression (e.g., no-treatment control group) ranged from 1.79 to 89.40% (Table 3).

The percent of cells with PD-L1 expression increased when treated with 0.6 µM IFNγ, which was used as a positive control, compared to the untreated controls. The percent increase in the staining of cells (with respect to the no-treatment controls) varied from 5.8% for SCC19 to 657.5% for SCC15 (Table 3).

Similarly, the percent of cells with PD-L1 expression increased when treated with 0.2, 2.0, or 20.0 µM HBD3 compared to the untreated controls. At 20.0 µM HBD3, the percent increase in the staining of cells (with respect to the no-treatment controls) varied from 7.9% for SCC19 to 109.0% for SCC15 (Table 3). HBD3 had a dose dependent increase in PD-L1 expression. The percent of SCC99 cells with PD-L1 expression increased with increasing concentrations of HBD3 (Figure 1g).

We performed Wilcoxon signed rank tests to compare differences between each of the treatment groups and the control group, as well as differences between the two treatment groups (Table 3). Treatment A (20 µM HBD3) was significantly elevated compared to the control group: *p* = 0.03 (one-sided test). Treatment B (0.6 µM IFNγ) was also significantly elevated compared to the control group: *p* = 0.03 (one-sided test). However, no significant difference was shown between Treatment A (20 µM HBD3) and Treatment B (0.6 µM IFNγ) (two-sided *p* = 1). Multiple comparisons were not adjusted.

## 3. Discussion

PD-L1 is an important immune checkpoint molecule in cancer pathogenesis regulating both tumor-intrinsic signaling and adaptive immunosuppression. It is induced by inflammatory and gamma-chain cytokines [15,16,17,20]. However, little is known about other factors in the oral cavity that also influence PD-L1 expression in SCC. Here we demonstrate that HBD3, a host defense peptide with diverse innate immune activities [29], is one of these factors. HBD3 is present in nasal mucus, saliva, and gingival crevicular fluid at concentrations as high as 6.2 mg/mL [43]. However, it is also present in abnormally high concentrations in the saliva of patients with oral cancers [44,45] likely associated with the intense inflammation associated with oral cancer in these individuals [44]. In our work, we have shown that HBD3 can bidirectionally regulate chemokine and cytokine responses [36,37]. Therefore, it is conceivable that elevated concentrations of HBD3, like that in oral cancer [44,45] can induce PD-L1 expression in SCC cells. To what extent it is involved in enhancing the suppression of tumor-specific T-cell activity and contributes to an immunosuppressive environment in the tissues of the oral cavity will have to be determined.

Recently, Ghosh and colleagues assessed the dysregulation of HBD3 in oral squamous cell carcinoma (OSCC) [46]. They noted that HBD3 is up regulated in OSCC tissues with respect to healthy oral mucosa [44] and is expressed in the cytoplasm of OSCC cells [47]. In HPV infected oral epithelial cells, oncogene E6 increases HBD3 mRNA and peptide expression and tumor suppressor p53 is inhibited by E6 and blocks HBD3 expression [45,46,48].

PD-L1 expression in cancers is used to predict a favorable outcome to PD-1 and PD-L1 immunotherapy treatments [49,50]. In recent studies, we predicted that PD-L1 induction stimuli in HNSCC cell lines SCC4, SCC15, and SCC25 were processed via ERK signaling pathways (via EGFR, BRAF-V600E (BRAF), MEK1/2 (MAP2K1, MAP2K2), ERK1/2 (MAPK3, MAPK1), and c-Jun (JUN)) [18,19]. We predicted high levels of PD-L1 expression is processed through STAT3 and ERK signaling pathways [51,52]. We predicted induction stimuli is also processed through the EGF receptor (Erb) signaling pathway (NRAS, PIK3CA, AKT, MTOR, STAT3) and the IFNγ pathway (IFNG, IFNGR1, STAT1, IRF1). Pathway signals converge to activation factors AP1, STAT1, STAT3, and IRF1 leading to transcription of PD-L1 genes.

HBD3 regulates many pathways including those that likely influence PD-L1 expression. Also in our work, we found that HBD3 at low concentrations attenuates pro-inflammatory agonist-induced chemokine and pro-inflammatory cytokine responses and at high concentrations, enhances agonist-induced chemokine and pro-inflammatory cytokine responses [36,37]. HBD3 binds to G-protein coupled receptor CCR6, which stimulates Gi and Gq respectively and activates the LYN/SYK/PLC/PKC-DAG pathway converging at the activation of AKT, NFKB, and NFAT. Future studies will determine whether HBD3-induced PD-L1 expression and IFNγ-induced PD-L1 expression share similar pathways.

## 4. Materials and Methods

### 4.1. PD-L1 Inducers

Human β defensin-3 (HBD3, catalog no. 300-52) was purchased from Peprotech, Rocky Hill, NJ, USA. The concentrations of HBD3 (0.2, 2.0, or 20.0 µM) used in this study were similar to those concentrations used in our previous studies [36] and those concentrations used in similar studies by others [53].

Recombinant human IFNγ (catalog no. 300-02) was purchased from Peprotech, Rocky Hill, NJ, USA. IFNγ is a well-known PD-L1 inducer [15] and was used as a positive control.

### 4.2. Cell Lines

Cell lines SCC4 (ATCC, Manassas, VA, USA), SCC15 (ATCC), SCC19 (University of Michigan), SCC25 (ATCC), and SCC99 (University of Michigan) were used in this study (Table 1). SCC4, SCC15, and SCC25, were from the oral cavity, while SCC19 and SCC99 were from the oropharynx. SCC4 [19], SCC15 [19], SCC25 [19], and SCC99 [42] have been previously genotyped. All cell lines have also been previously authenticated [10] using the ANSI Standard (ASN-0002) Authentication of Human Cell Lines: Standardization of STR Profiling by the ATCC Standards Development Organization.

Cell line SCC4 was grown in complete Dulbecco’s Modified Eagle’s Medium: F-12 (DMEM: F-12) containing 2 mM L-glutamine, 1% nonessential amino acids (ATCC), 400 ng/mL hydrocortisone (Sigma-Aldrich Corp., St. Louis, MO, USA), 100 units/mL penicillin (Life Technologies, Madison, WI, USA), 100 units/mL streptomycin (Life Technologies), and 10% fetal bovine serum (ATCC) [19].

Cell lines SCC15 and SCC25 were grown in complete Lymphocyte Growth Media-3 (LGM-3) (Lonza, Walkersville, MD, USA), 100 units/mL penicillin (Life Technologies), 100 units/mL streptomycin (Life Technologies), and 10% fetal bovine serum (ATCC) [19].

Cell lines SCC19 and SCC99 were grown in Dulbecco’s Modified Eagle’s Medium (DMEM) containing 2 mM L-glutamine, 1% nonessential amino acids (ATCC), 100 units/mL penicillin (Life Technologies), 100 units/mL streptomycin (Life Technologies), and 10% fetal bovine serum (ATCC).

### 4.3. Immunohistochemistry

A double-sandwich ELISA (MyBioSource, Inc., San Diego, CA, USA) was used to detect human papillomavirus (HPV) antigen tissue culture media and cell lysates as previously described [10].

Immunohistochemistry (IHC) was used to detect p16^Ink4a^ in cell lines as previously described [10]. Cell pellets were fixed in 10% neutral buffered formalin, immobilized in agar, embedded in paraffin, and sectioned. Sections were deparaffinized and stained with an antibody to p16^Ink4a^. Squamous cell carcinoma of the uterus was used as a positive tissue control.

### 4.4. Induction of PD-L1 Expression

SCC cells (5.0 × 10^4^ viable cells) were incubated without and with 0.6 µM IFNγ or 0.2, 2.0, or 20.0 µM HBD3 for 24 h. The number of cells expressing PD-L1 was determined by flow cytometry as described below in our laboratory [19].

### 4.5. Detection of Cells Expressing PD-L1 by Flow Cytometry

Cells were first stained with Live/Dead Fixable Green Dead Cell Stain (BD Biosciences, San Jose, CA, USA), then stained with anti-human APC-CD274 (563741 PD-L1, BD Pharmingen, San Jose, CA), and then examined using an LSR II Violet Flow Cytometer (BD Biosciences). Cells stained with an isotype control (APC mouse IgG1 κ, BD Pharmingen, San Jose, CA, USA) served as a control to account for any non-specific binding. Cells not stained were also included as controls. Flow cytometric data was analyzed using FlowJo software (Tree Star, Inc., Ashland, OR, USA) [19].

### 4.6. Statistical Analysis

Wilcoxon signed rank tests were performed to compare between each of the treatment groups and the control group, as well as between the two treatment groups. Treatment A was 20.0 µM HBD3 was and Treatment B was 0.6 µM IFNγ. Multiple comparisons were not adjusted.

## 5. Conclusions

In conclusion, HBD3 is thought to have a role in the pathogenesis of oral cancers [44,45,46]. Here we show that HBD3 increases the number of HNSCC cells expressing PD-L1. However, to what extent HBD3 contributes to HNSCC pathogenesis by inducing PD-L1 expression in tumors will have to be determined.

## Figures and Tables

**Table 1:** Head and neck squamous cell carcinomas (HNSCC) cell lines used in this study. SCC4, SCC15, and SCC25 are from the oral cavity, while SCC19 and SCC99 are from the oropharynx. The basic characteristics for each cell line are listed below.

Cell Line	Sex	Anatomical Site (Oral Cavity)	TNM Stage
SCC4	M	Tongue	T3N0M0
SCC15	M	Tongue	T4N1M0
SCC25	M	Tongue	T2N1
UM-SCC19	M	Oropharynx/base of tongue	T2N1M0
UM-SCC99	M	Oropharynx	T3N0M0

Cell lines were derived from HNSCC and SCC malignant tumors [19,42]. (TNM, where T describes the size of the primary tumor, N describes the nearby lymph nodes, and M describes the metastasis).

**Table 2:** Programmed death-ligand 1 (PD-L1) concentrations (pg/mL) in cell lysates of SCC4, SCC15, SCC19, SCC25, and SCC99.

Cell Line	N	Mean	SD	Minimum	Maximum	Median
SCC4	3	241.40	58.79	207.17	309.28	207.75
SCC15	3	193.54	93.45	125.91	300.17	154.53
SCC19	3	92.65	15.09	79.67	109.21	89.08
SCC25	3	387.59	135.59	279.68	539.79	343.29
SCC99	3	206.16	47.72	173.10	260.87	184.52

Values (pg/mL) were from our recent work used as the basis for the article by Bates et al. [10].

**Table 3:** Flow cytometry analysis showing the percent of SCC4, SCC15, SCC19, SCC25, and SCC99 cells with PD-L1 expression after treatments with 20.0 µM human β-defensin 3 (HBD3) (test) or 0.6 µM IFNγ (control). The percent increase in staining of cells with PD-L1 expression treated with 0.6 µM IFNγ or 20.0 µM HBD3 over the no-treatment control cells is included.

Squamous Cell Carcinoma (SCC) Cell Lines	Unstained Cell Controls (%)	Isotype Ab Treated Cell Controls (%)	No-Treatment Control Cells (%)	0.6 µM IFNγ Treated Cells (%)	20.0 µM HBD3 Treated Cells (%)
SCC4	0.11 ^a^	0.30	1.79	5.90	2.64
			(0.0) ^b^	(229.6)	(47.5)
SCC15	0.05	0.04	5.34	40.45	11.16
			(0.0)	(657.5)	(109.0)
SCC19	0.10	0.09	89.40	94.60	96.50
			(0.0)	(5.8)	(7.9)
SCC25	0.04	0.16	12.28	13.68	16.75
			(0.0)	(11.4)	(36.4)
SCC99	0.02	0.29	40.30	63.60	70.40
			(0.0)	(57.8)	(74.7)

^a^ The percent of cells with PD-L1 expression. ^b^ The percent increase of cells with PD-L1 expression treated with 0.6 µM IFNγ or 20.0 µM HBD3 over the no-treatment control cells.
